# Exploring the Use and Implications of AI in Sexual and Reproductive Health and Rights: Protocol for a Scoping Review

**DOI:** 10.2196/53888

**Published:** 2024-04-09

**Authors:** Tigest Tamrat, Yu Zhao, Denise Schalet, Shada AlSalamah, Sameer Pujari, Lale Say

**Affiliations:** 1 UNDP/UNFPA/UNICEF/WHO/World Bank Special Programme of Research, Development and Research Training in Human Reproduction Department of Sexual and Reproductive Health and Research World Health Organization Geneva Switzerland; 2 Department of Digital Health and Innovations Science Division World Health Organization Geneva Switzerland; 3 Information Systems Department College of Computer and Information Sciences King Saud University Riyadh Saudi Arabia

**Keywords:** artificial intelligence, AI, sexual health, reproductive health, maternal health, gender, machine learning, natural language processing, review, systematic documentation, protocol, scoping review, electronic database, technical consultation, intervention, methodology, qualitative, World Health Organization, WHO, decision-making

## Abstract

**Background:**

Artificial intelligence (AI) has emerged as a transformative force across the health sector and has garnered significant attention within sexual and reproductive health and rights (SRHR) due to polarizing views on its opportunities to advance care and the heightened risks and implications it brings to people’s well-being and bodily autonomy. As the fields of AI and SRHR evolve, clarity is needed to bridge our understanding of how AI is being used within this historically politicized health area and raise visibility on the critical issues that can facilitate its responsible and meaningful use.

**Objective:**

This paper presents the protocol for a scoping review to synthesize empirical studies that focus on the intersection of AI and SRHR. The review aims to identify the characteristics of AI systems and tools applied within SRHR, regarding health domains, intended purpose, target users, AI data life cycle, and evidence on benefits and harms.

**Methods:**

The scoping review follows the standard methodology developed by Arksey and O’Malley. We will search the following electronic databases: MEDLINE (PubMed), Scopus, Web of Science, and CINAHL. Inclusion criteria comprise the use of AI systems and tools in sexual and reproductive health and clear methodology describing either quantitative or qualitative approaches, including program descriptions. Studies will be excluded if they focus entirely on digital interventions that do not explicitly use AI systems and tools, are about robotics or nonhuman subjects, or are commentaries. We will not exclude articles based on geographic location, language, or publication date. The study will present the uses of AI across sexual and reproductive health domains, the intended purpose of the AI system and tools, and maturity within the AI life cycle. Outcome measures will be reported on the effect, accuracy, acceptability, resource use, and feasibility of studies that have deployed and evaluated AI systems and tools. Ethical and legal considerations, as well as findings from qualitative studies, will be synthesized through a narrative thematic analysis. We will use the PRISMA-ScR (Preferred Reporting Items for Systematic Reviews and Meta-Analyses Extension for Scoping Reviews) format for the publication of the findings.

**Results:**

The database searches resulted in 12,793 records when the searches were conducted in October 2023. Screening is underway, and the analysis is expected to be completed by July 2024.

**Conclusions:**

The findings will provide key insights on usage patterns and evidence on the use of AI in SRHR, as well as convey key ethical, safety, and legal considerations. The outcomes of this scoping review are contributing to a technical brief developed by the World Health Organization and will guide future research and practice in this highly charged area of work.

**Trial Registration:**

OSF Registries osf.io/ma4d9; https://osf.io/ma4d9

**International Registered Report Identifier (IRRID):**

PRR1-10.2196/53888

## Introduction

Artificial intelligence (AI) refers to the development of algorithms, processes, machines, and computer programs capable of performing automated tasks, without the programming of each step explicitly by a human [1,2]. Advances in computing processing combined with the amassing of data through digital tools, particularly with increases in the penetration of mobile devices, have propelled the field of AI within health [3-6]. AI includes approaches such as machine learning, in which statistical and mathematical modeling techniques are used to define and analyze data. This can also be applied through natural language processing to analyze text-based data and signal processing for audio, images, and videos [6,7]. The use of AI varies across health fields, with radiology and pathology being the dominant areas where machine learning has been leveraged to optimize the processing of large volumes of medical imaging data [6,7]. Recent advancements in AI, particularly generative AI and large language models, have expanded the ability to address a diverse set of health needs.

The field of sexual and reproductive health encompasses health domains, such as family planning and fertility care, maternal health, sexually transmitted infections (STIs), safe abortion care, sexual health and well-being, and gender-based violence [8,9]. It is underpinned by broader principles of bodily autonomy, human rights, women’s empowerment, and gender equality, encapsulated as sexual and reproductive health and rights (SRHR) [10]. Considering the perceived sensitivity and often politicization of these health topics beyond the influence of technology, the use of AI in SRHR is an emerging area with both great potential and justifiable concerns. Individuals’ desires for anonymity in seeking sexual and reproductive health services positions AI systems and tools, such as conversational agents, as critical conduits for expanding access to information and care [11-13]. Furthermore, shortfalls in human resources and the need for targeted health interventions, such as in the areas of maternal health care and management of STIs, serve as key issues that have the potential to be addressed through the predictive capabilities offered by AI [14]. In addition, the use of AI to power “software as a medical device” [15] presents an opportunity to leapfrog access to diagnostic devices, such as ultrasounds [16] and blood pressure equipment [17]. This has also been seen with the emergence of contraceptive and fertility software applications [18,19], which are increasingly securing regulatory approvals from national authorities [20,21].

The convergence of AI in SRHR also presents heightened risks and implications. This is especially evident as the use of underlying health data raises concerns about infringements on women’s health and bodily autonomy, as well as the potential misuse of this technology for surveillance of populations in vulnerable situations. Furthermore, existing challenges in digital health implementation [22], including biases, limited inclusivity, and gender disparities, could be exacerbated with the introduction of AI [6,23-25]. As such, the intersection of AI and SRHR raises nuanced implications and warrants close examination of the current landscape to highlight the evidence base and document the ethical, regulatory, and human rights considerations. This scoping review will build on the general literature of AI in health and focus specifically on how AI is being used within SRHR, including evidence on effect and related considerations to inform a comprehensive understanding of the state of the field.

## Methods

### Overview

We will conduct a scoping review using the established methodological framework of Arksey and O’Malley [26] that includes (1) identifying the research question; (2) identifying relevant studies; (3) study selection; (4) charting the data; and (5) collating, summarizing, and reporting the results. Considering this is an emerging area of research, we will also include a stakeholder consultation to inform the discussion. We will follow the PRISMA-ScR (Preferred Reporting Items for Systematic Reviews and Meta-Analyses Extension for Scoping Reviews) format for the publication of the findings [27].

### Stage 1: Identifying Research Questions

This scoping review aims to explore the range of ways in which AI is being applied in SRHR and synthesize the key considerations to ensure its effective, safe, and ethical use. The research questions for this review include the following:

- Patterns and characteristics of use: How are AI systems and tools being applied to SRHR, in terms of health domains, intended purpose (eg, screening, counseling, forecasting), target users, and AI data life cycle?
- Evidence on harms and benefits: What are the effect, acceptability, feasibility, resource use, and implications on gender, equity, and rights of AI systems and tools used in SRHR?
- Ethical, legal, and safety implications: What are the ethical, legal, and safety considerations specific to the use of AI systems and tools in SRHR?

### Stage 2: Identifying Relevant Studies

To identify relevant studies for inclusion, we will search the following electronic databases: MEDLINE (PubMed), Scopus, Web of Science, and CINAHL. In addition, we will use citation searching from relevant articles to identify sources that may not have been retrieved in our original search.

The search strategy will be a combination of constructs related to AI and SRHR. An overview of search terms is presented in Multimedia Appendix 1. We will leverage search strategies and Medical Subject Headings (MeSH) terms used in other scoping reviews focused on AI in health care [28-32]. Considering this is an emerging topic, we will not set a start date for the search.

### Stage 3: Study Selection

We will conduct a 2-step abstract and title screening following the eligibility criteria in Textbox 1. We will use Covidence, a standard web-based screening and extraction tool, to manage the screening process.

We will not exclude manuscripts based on geographic location, language, or publication date. Commentaries, opinions, and editorials that do not have an underlying empirical basis will be excluded to adjust for potential subjectivity and bias. In addition to using Covidence, we will search the citations of included studies to identify relevant articles that may have been overlooked in our initial search.

All references will be collated into a single reference manager (EndNote), where duplicate entries will be removed. Articles will be screened independently by 2 coauthors. Identified discrepancies will be resolved through discussion, and, if needed, escalated to a coauthor for arbitration.

**Inclusion and exclusion criteria.**

**Inclusion criteria**

- Studies reporting on artificial intelligence (AI) applications to aspects of sexual and reproductive health and rights
- Clearly described methodology; we will include quantitative studies, qualitative studies, and program evaluations and descriptions

**Exclusion criteria**

- Focus exclusively on digital interventions (eg, traditional text messaging or targeted client communication) that do not explicitly use AI systems and tools
- Focus on health care robotics or nonhuman studies
- Commentaries, opinion pieces, and editorials

### Stage 4: Charting the Data

We will develop a data extraction sheet to standardize the retrieval of information in alignment with the research questions (Table 1). The data extraction form will be developed by adapting common themes from other scoping reviews on AI [31,33], as well as reviewing requirements from digital health reporting checklists [34,35], information on the intervention, including how AI was applied to SRHR, the specific SRHR domains of interest, population, geographic coverage, implementation challenges, outcomes, and ethical and legal considerations.

**Table 1:** Categories for data extraction.

Category	Illustrative information to be extracted	Relevant research questions
Article information	Article title Author(s) Year Country Setting or context (facility, community, home or self, research) Aim or objectives Study design or methodology	Patterns of use
SRHR^a^ health domain	To be selected from a predefined list of common SRHR areas and expanded on accordingly Examples include family planning or contraception counseling and provision, fertility care, safe abortion, sexually transmitted infections, antenatal care, postnatal care, etc	Patterns of use
Targeted population	Will be selected from a predefined list and expanded accordingly. Examples include health workers, health system managers, individuals or health service users	Patterns of use
Intended purpose of AI^b^	The intended purposes for using AI: Health information, education, and promotion Screening and diagnostics Clinical care and management Personal health monitoring Forecasting health trends Health systems management Research and drug development	Patterns of use
AI life cycle	Stages of AI development and evaluation [36]: Data creation Data acquisition Model development Model evaluation Model deployment	Patterns of use
Algorithm development and models	Predictive or generative models Documentation of algorithm development and training process Presence of regulatory approval	Patterns of use
Outcomes and findings	Outcomes based on the Evidence to Decision framework [37], which includes effect, accuracy, acceptability, feasibility, equity, resource considerations, gender, equity, and rights Key themes and findings Implementation challenges Lessons learned Unintended consequences, risks, and implications Approaches used to mitigate unintended consequences	Evidence on harms and benefits
Implications	Legal implications Ethical implications	Ethical and legal implications

^a^SRHR: sexual and reproductive health and rights.

^b^AI: artificial intelligence.

### Stage 5: Collating, Summarizing, and Reporting the Results

We will collate and summarize information from the data extraction sheets. The results will present the general patterns of use of AI in SRHR, including the specific health domains for which AI is being applied, the intended purpose of the AI system and tools, and maturity within the AI life cycle. We will use established frameworks, including the continuum of care and SRHR conceptual model for categorizing health domains [8,9] and the AI life cycle to map the maturity of AI systems and tools in use [36].

To assess evidence on harms and benefits, studies will first be tagged based on their AI life cycle [36] to distinguish between interventions that are in the early stages of model development and validation versus those that have been implemented and evaluated. We will extract outcomes on the subset of studies identified as being in the model deployment and evaluation phases of the AI life cycle [36]. The type of outcome data to be extracted will be guided by the domains of the Evidence to Decision framework, including accuracy, effect, acceptability, feasibility, resource use, and gender equity and rights [37]. Where possible, we will pool findings across similar outcome measures and present a summary of findings on outcomes related to accuracy and effect. We will conduct a thematic analysis on ethical, legal, and safety considerations, as well as on findings from qualitative studies.

## Results

The database searches described above resulted in 12,793 records when the searches were conducted in October 2023. Screening is underway, and the analysis is expected to be completed by July 2024.

## Discussion

Building on the broader literature on AI and health, findings from this review will provide fundamental insights into the usage patterns, implications, and evidence related to AI in SRHR, as well as highlight key considerations and risks. A key strength of this review is the composition of a multidisciplinary authorship team to ensure both the technological and SRHR perspectives are appropriately reflected. Moreover, this scoping review serves as a foundational resource to inform a technical brief developed by the World Health Organization [38] and to guide future research and practice. One limitation of this review is its focus on empirical studies, in which insights from the gray literature and AI consumer products may be overlooked if they are not reflected in the peer-reviewed literature. However, due to the high number of records in the search, we believe this scoping review can reliably provide a comprehensive overview of usage patterns and key implications.

The intersection of AI and SRHR has been fraught with concerns, particularly related to infringements on women’s reproductive health choices and the exacerbation of gender biases and inequity [39]. Meanwhile, technological advances hold great promise for overcoming longstanding challenges in access to and provision of SRHR services. This systematic analysis of AI in SRHR seeks to facilitate clarity and nuanced discussion in this highly charged field and direct efforts toward responsibly and meaningfully harnessing of AI to address SRHR needs and the broader goals of universal health coverage.

## Appendix

Multimedia Appendix 1 Search strategy.

## Abbreviations

AI: artificial intelligence
PRISMA-ScR: Preferred Reporting Items for Systematic Reviews and Meta-Analyses Extension for Scoping Reviews
MeSH: Medical Subject Headings
SRHR: sexual and reproductive health and rights
STI: sexually transmitted infection

## Data Availability

Access to the Covidence database can be made available after the publication of findings by emailing srhhrp@who.int and through a mutually signed agreement.
