# Supplementary material for: Exploring the Use and Implications of AI in Sexual and Reproductive Health and Rights: Protocol for a Scoping Review
Source: JMIR Res Protoc. 2024 Apr 9;13:e53888. doi: 10.2196/53888 (PMC11040437; doi:10.2196/53888)
Supplement: Multimedia Appendix 1 [file resprot_v13i1e53888_app1.docx]

**Artificial intelligence and sexual and reproductive health and rights: Protocol for scoping review**

**Appendix 1. Search strategy**

Databases: PubMed, Scopus, Web of Science, and CINAHL

Date Run: 10/17/2023

Total of all Search Results: 22,985

Total Unique Citations after Deduplication: 12,793

PubMed via the U.S. National Library of Medicine

| **Concept** | **Search Terms** | **Results** |
| --- | --- | --- |
| #1 Sexual and reproductive health | ("Sexual Health"[Mesh] OR "Reproductive Health"[Mesh] OR "Reproductive Health Services"[Mesh] OR "sexual health"[tiab] OR "reproductive health"[tiab] OR "reproductive medicine"[tiab] OR "women’s health"[tiab]) OR ("Reproductive Rights"[Mesh] OR "Family Planning Services"[Mesh] OR "Contraception"[Mesh] OR "Contraceptive Devices"[Mesh] OR "Abortion, Induced"[Mesh] OR "Gender-Based Violence"[Mesh] OR "reproductive rights"[tiab] OR "reproduction rights"[tiab] OR "sexual rights"[tiab] OR "reproductive freedom"[tiab] OR "sexual freedom"[tiab] OR "gender-based violence"[tiab] OR "gender violence"[tiab] OR "sexual violence"[tiab] OR "sexual coercion"[tiab] OR "family planning"[tiab] OR "safe sex"[tiab] OR contraception[tiab] OR contraceptive[tiab] OR contraceptives[tiab] OR "birth control"[tiab] OR abortion[tiab] OR abortions[tiab]) OR ("Pregnancy"[Mesh] OR "Pregnant Women"[Mesh] OR "Maternal Health"[Mesh] OR obstetrics[tiab] OR pregnant[tiab] OR pregnancy[tiab] OR pregnancies[tiab] OR "maternal health"[tiab] OR "maternal healthcare"[tiab] OR prenatal[tiab] OR perinatal[tiab] OR antenatal[tiab] OR peripartum[tiab] OR antepartum[tiab] OR postpartum[tiab] OR postnatal[tiab] OR fertility[tiab] OR infertility[tiab] OR "reproductive sterility"[tiab] OR subfertility[tiab]) OR ("Uterine Cervical Neoplasms"[Mesh] OR "cervical neoplasm"[tiab] OR "cervical neoplasms"[tiab] OR "cervical cancer"[tiab] OR "cervix cancer"[tiab]) OR ("Human Papillomavirus Viruses"[Mesh] OR "Papillomavirus Infections"[mesh] OR "Human Papilloma Virus"[tiab] OR "Human Papillomavirus"[tiab] OR HPV[tiab]) OR (HIV[Mesh] OR HIV[tiab] OR "Human Immunodeficiency Virus"[tiab] OR AIDS[tiab] OR "Acquired Immune Deficiency Syndrome Virus"[tiab] OR "Acquired Immunodeficiency Syndrome Virus"[tiab]) OR ("Sexually Transmitted Diseases"[Majr] OR "sexually transmitted disease"[tiab] OR "sexually transmitted diseases"[tiab] OR "sexually transmitted infection"[tiab] OR "sexually transmitted infections"[tiab] OR STD[tiab] OR STI[tiab] OR STIs[tiab] OR STDs[tiab] OR "venereal disease"[tiab] OR "venereal diseases"[tiab]) | 2,175,872 |
| #2 Artificial intelligence | ("Artificial Intelligence"[Mesh] OR "Machine Learning"[Mesh] OR "Neural Networks, Computer"[Mesh] OR "computer reasoning"[tiab] OR "machine learning"[tiab] OR "artificial intelligence"[tiab] OR "computational intelligence"[tiab] OR "machine intelligence"[tiab] OR "computer learning"[tiab] OR "predictive analytics"[tiab] OR "gradient boosted decision"[tiab] OR "support vector machine"[tiab] OR "random forest model"[tiab] OR "neural network"[tiab] OR "neural networks"[tiab] OR "manifold learning"[tiab] OR "deep learning"[tiab] OR "active learning"[tiab] OR "learning algorithm"[tiab] OR "learning algorithms"[tw] OR "AI algorithms"[tiab] OR AI[ti] OR "natural language processing"[tiab] OR "natural language understanding"[tiab] OR "software as a medical device"[tiab] OR chatbot[tiab] OR "conversational agent"[tiab] OR "large language model"[tiab] OR "large language models"[tiab] OR LLMs[tiab] OR "sentiment analysis"[tiab] OR "quantitative distribution"[tiab] OR "frequency distribution"[tiab]) | 337,622 |
| #3 Combined concepts | #1 AND #2 | 8,472 |
| #4 Combined with filters | #3 NOT ("Robotics"[MAJR] OR "Comment" [Publication Type] OR "Editorial" [Publication Type] OR "Letter" [Publication Type] OR "Retracted Publication" [Publication Type]) NOT (animals[Mesh] NOT humans[Mesh]) | 7,010 |

Scopus via Elsevier

| **Concept** | **Search Terms** | **Results** |
| --- | --- | --- |
| #1 Sexual and reproductive health | (TITLE-ABS("sexual health") OR TITLE-ABS("reproductive health") OR TITLE-ABS("reproductive medicine") OR TITLE-ABS("women’s health") OR TITLE-ABS("reproductive rights") OR TITLE-ABS("reproduction rights") OR TITLE-ABS("sexual rights") OR TITLE-ABS("reproductive freedom") OR TITLE-ABS("sexual freedom") OR TITLE-ABS("gender-based violence") OR TITLE-ABS("gender violence") OR TITLE-ABS("sexual violence") OR TITLE-ABS("sexual coercion") OR TITLE-ABS("family planning") OR TITLE-ABS("safe sex") OR TITLE-ABS(contraception) OR TITLE-ABS(contraceptive) OR TITLE-ABS(contraceptives) OR TITLE-ABS("birth control") OR TITLE-ABS(abortion) OR TITLE-ABS(abortions) OR TITLE-ABS(obstetrics) OR TITLE-ABS(pregnant) OR TITLE-ABS(pregnancy) OR TITLE-ABS(pregnancies) OR TITLE-ABS("maternal health") OR TITLE-ABS("maternal healthcare") OR TITLE-ABS(prenatal) OR TITLE-ABS(perinatal) OR TITLE-ABS(antenatal) OR TITLE-ABS(peripartum) OR TITLE-ABS(antepartum) OR TITLE-ABS(postpartum) OR TITLE-ABS(postnatal) OR TITLE-ABS(fertility) OR TITLE-ABS(infertility) OR TITLE-ABS("reproductive sterility") OR TITLE-ABS(subfertility) OR TITLE-ABS("cervical neoplasm") OR TITLE-ABS("cervical neoplasms") OR TITLE-ABS("cervical cancer") OR TITLE-ABS("cervix cancer") OR TITLE-ABS("Human Papilloma Virus") OR TITLE-ABS("Human Papillomavirus") OR TITLE-ABS(HPV) OR TITLE-ABS(HIV) OR TITLE-ABS("Human Immunodeficiency Virus") OR TITLE-ABS("Acquired Immune Deficiency Syndrome Virus") OR TITLE-ABS("Acquired Immunodeficiency Syndrome Virus") OR TITLE-ABS("sexually transmitted disease") OR TITLE-ABS("sexually transmitted diseases") OR TITLE-ABS("sexually transmitted infection") OR TITLE-ABS("sexually transmitted infections") OR TITLE-ABS("venereal disease") OR TITLE-ABS("venereal diseases")) | 1,952,610 |
| #2 Artificial intelligence | (TITLE-ABS("computer reasoning") OR TITLE-ABS("machine learning") OR TITLE-ABS("artificial intelligence") OR TITLE-ABS("computational intelligence") OR TITLE-ABS("machine intelligence") OR TITLE-ABS("computer learning") OR TITLE-ABS("predictive analytics") OR TITLE-ABS("gradient boosted decision") OR TITLE-ABS("support vector machine") OR TITLE-ABS("random forest model") OR TITLE-ABS("neural network") OR TITLE-ABS("neural networks") OR TITLE-ABS("manifold learning") OR TITLE-ABS("deep learning") OR TITLE-ABS("active learning") OR TITLE-ABS("learning algorithm") OR TITLE-ABS-KEY("learning algorithms") OR TITLE-ABS("AI algorithms") OR TITLE(AI) OR TITLE-ABS("natural language processing") OR TITLE-ABS("natural language understanding") OR TITLE-ABS("software as a medical device") OR TITLE-ABS(chatbot) OR TITLE-ABS("conversational agent") OR TITLE-ABS("large language model") OR TITLE-ABS("large language models") OR TITLE-ABS(LLMs) OR TITLE-ABS("sentiment analysis") OR TITLE-ABS("quantitative distribution") OR TITLE-ABS("frequency distribution")) | 1,633,348 |
| #3 Combined concepts | #1 AND #2 | 8,937 |
| #4 Filter Robotics/Animals | ( TITLE ( "Robotics" )  OR  TITLE ( "Robot" )  OR  TITLE ( "Rat" )  OR  TITLE ( "Rats" )  OR  TITLE ( "Mouse" )  OR  TITLE ( "Mice" )) |  |
| #5 | #3 AND NOT #4 | 8,756 |
| #6 Document types to exclude | (DOCTYPE(ch) OR DOCTYPE(cr) OR DOCTYPE(no) OR DOCTYPE(bk) OR DOCTYPE(ed) OR DOCTYPE(er) OR DOCTYPE(sh) OR DOCTYPE(le) OR DOCTYPE(tb)) | 10,525,803 |
| #7 | #5 AND NOT #6 | 8,196 |

Web of Science via Clarivate

| **Concept** | **Search Terms** | **Results** |
| --- | --- | --- |
| #1 Sexual and reproductive health | ((TI="sexual health" OR AB="sexual health") OR (TI="reproductive health" OR AB="reproductive health") OR (TI="reproductive medicine" OR AB="reproductive medicine") OR (TI="women’s health" OR AB="women’s health") OR (TI="reproductive rights" OR AB="reproductive rights") OR (TI="reproduction rights" OR AB="reproduction rights") OR (TI="sexual rights" OR AB="sexual rights") OR (TI="reproductive freedom" OR AB="reproductive freedom") OR (TI="sexual freedom" OR AB="sexual freedom") OR (TI="gender-based violence" OR AB="gender-based violence") OR (TI="gender violence" OR AB="gender violence") OR (TI="sexual violence" OR AB="sexual violence") OR (TI="sexual coercion" OR AB="sexual coercion") OR (TI="family planning" OR AB="family planning") OR (TI="safe sex" OR AB="safe sex") OR (TI=contraception OR AB=contraception) OR (TI=contraceptive OR AB=contraceptive) OR (TI=contraceptives OR AB=contraceptives) OR (TI="birth control" OR AB="birth control") OR (TI=abortion OR AB=abortion) OR (TI=abortions OR AB=abortions) OR (TI=obstetrics OR AB=obstetrics) OR (TI=pregnant OR AB=pregnant) OR (TI=pregnancy OR AB=pregnancy) OR (TI=pregnancies OR AB=pregnancies) OR (TI="maternal health" OR AB="maternal health") OR (TI="maternal healthcare" OR AB="maternal healthcare") OR (TI=prenatal OR AB=prenatal) OR (TI=perinatal OR AB=perinatal) OR (TI=antenatal OR AB=antenatal) OR (TI=peripartum OR AB=peripartum) OR (TI=antepartum OR AB=antepartum) OR (TI=postpartum OR AB=postpartum) OR (TI=postnatal OR AB=postnatal) OR (TI=fertility OR AB=fertility) OR (TI=infertility OR AB=infertility) OR (TI="reproductive sterility" OR AB="reproductive sterility") OR (TI=subfertility OR AB=subfertility) OR (TI="cervical neoplasm" OR AB="cervical neoplasm") OR (TI="cervical neoplasms" OR AB="cervical neoplasms") OR (TI="cervical cancer" OR AB="cervical cancer") OR (TI="cervix cancer" OR AB="cervix cancer") OR TS="Papillomavirus Infections" OR (TI="Human Papilloma Virus" OR AB="Human Papilloma Virus") OR (TI="Human Papillomavirus" OR AB="Human Papillomavirus") OR (TI=HPV OR AB=HPV) OR (TI=HIV OR AB=HIV) OR (TI="Human Immunodeficiency Virus" OR AB="Human Immunodeficiency Virus") OR (TI="Acquired Immune Deficiency Syndrome Virus" OR AB="Acquired Immune Deficiency Syndrome Virus") OR (TI="Acquired Immunodeficiency Syndrome Virus" OR AB="Acquired Immunodeficiency Syndrome Virus") OR (TI="sexually transmitted disease" OR AB="sexually transmitted disease") OR (TI="sexually transmitted diseases" OR AB="sexually transmitted diseases") OR (TI="sexually transmitted infection" OR AB="sexually transmitted infection") OR (TI="sexually transmitted infections" OR AB="sexually transmitted infections") OR (TI="venereal disease" OR AB="venereal disease") OR (TI="venereal diseases" OR AB="venereal diseases")) | [2,169,933](https://www.webofscience.com/wos/alldb/summary/7b280529-852f-4f24-82f4-274a51b69f5c-ac86735a/relevance/1) |
| #2 Artificial intelligence | ((TI="computer reasoning" OR AB="computer reasoning") OR (TI="machine learning" OR AB="machine learning") OR (TI="artificial intelligence" OR AB="artificial intelligence") OR (TI="computational intelligence" OR AB="computational intelligence") OR (TI="machine intelligence" OR AB="machine intelligence") OR (TI="computer learning" OR AB="computer learning") OR (TI="predictive analytics" OR AB="predictive analytics") OR (TI="gradient boosted decision" OR AB="gradient boosted decision") OR (TI="support vector machine" OR AB="support vector machine") OR (TI="random forest model" OR AB="random forest model") OR (TI="neural network" OR AB="neural network") OR (TI="neural networks" OR AB="neural networks") OR (TI="manifold learning" OR AB="manifold learning") OR (TI="deep learning" OR AB="deep learning") OR (TI="active learning" OR AB="active learning") OR (TI="learning algorithm" OR AB="learning algorithm") OR TS="learning algorithms" OR (TI="AI algorithms" OR AB="AI algorithms") OR TI=AI OR (TI="natural language processing" OR AB="natural language processing") OR (TI="natural language understanding" OR AB="natural language understanding") OR (TI="software as a medical device" OR AB="software as a medical device") OR (TI=chatbot OR AB=chatbot) OR (TI="conversational agent" OR AB="conversational agent") OR (TI="large language model" OR AB="large language model") OR (TI="large language models" OR AB="large language models") OR (TI=LLMs OR AB=LLMs) OR (TI="sentiment analysis" OR AB="sentiment analysis") OR (TI="quantitative distribution" OR AB="quantitative distribution") OR (TI="frequency distribution" OR AB="frequency distribution")) | [1,228,462](https://www.webofscience.com/wos/alldb/summary/f6815b38-32e4-41ff-963c-1c87de0af3e7-ac8680d9/relevance/1) |
| #3 Combined concepts | #1 AND #2 | [8,046](https://www.webofscience.com/wos/alldb/summary/edb3d269-b764-40b0-80f4-7077749eb91a-ac868a44/relevance/1) |
| #4 Combined with filters | #3 NOT ( (TI=Robotics) OR (TI=Robot) OR (TI=Rat) OR (TI=Rats) OR (TI=Mouse) OR (TI=Mice)) | [7,857](https://www.webofscience.com/wos/alldb/summary/746ba53f-4b17-403e-83dd-72927e20709b-ac869266/relevance/1) |
| #5 | Exclude Filter: Document Types: Proceeding Paper OR Abstract OR Editorial Material OR Book Chapters OR Letter OR Book Review OR Meeting OR Note OR Retracted Publication | 6305 |

CINAHL Plus via EBSCO

| **Concept** | **Search Terms** | **Results** |
| --- | --- | --- |
| #1 Sexual and reproductive health | ((MH "Sexual Health+") OR (MH "Reproductive Health+") OR (TI "sexual health" OR AB "sexual health") OR (TI "reproductive health" OR AB "reproductive health") OR (TI "reproductive medicine" OR AB "reproductive medicine") OR (TI "women’s health" OR AB "women’s health")) OR ((MH "Reproductive Rights+") OR (MH "Family Planning+") OR (MH Contraception+) OR (MH "Contraceptive Agents+") OR (MH "Abortion, Induced+") OR (MH "Gender-Based Violence+") OR (TI "reproductive rights" OR AB "reproductive rights") OR (TI "reproduction rights" OR AB "reproduction rights") OR (TI "sexual rights" OR AB "sexual rights") OR (TI "reproductive freedom" OR AB "reproductive freedom") OR (TI "sexual freedom" OR AB "sexual freedom") OR (TI "gender-based violence" OR AB "gender-based violence") OR (TI "gender violence" OR AB "gender violence") OR (TI "sexual violence" OR AB "sexual violence") OR (TI "sexual coercion" OR AB "sexual coercion") OR (TI "family planning" OR AB "family planning") OR (TI "safe sex" OR AB "safe sex") OR (TI contraception OR AB contraception) OR (TI contraceptive OR AB contraceptive) OR (TI contraceptives OR AB contraceptives) OR (TI "birth control" OR AB "birth control") OR (TI abortion OR AB abortion) OR (TI abortions OR AB abortions)) OR ((MH Pregnancy+) OR (MH "Expectant Mothers+") OR (MH "Maternal Health Services+") OR (TI obstetrics OR AB obstetrics) OR (TI pregnant OR AB pregnant) OR (TI pregnancy OR AB pregnancy) OR (TI pregnancies OR AB pregnancies) OR (TI "maternal health" OR AB "maternal health") OR (TI "maternal healthcare" OR AB "maternal healthcare") OR (TI prenatal OR AB prenatal) OR (TI perinatal OR AB perinatal) OR (TI antenatal OR AB antenatal) OR (TI peripartum OR AB peripartum) OR (TI antepartum OR AB antepartum) OR (TI postpartum OR AB postpartum) OR (TI postnatal OR AB postnatal) OR (TI fertility OR AB fertility) OR (TI infertility OR AB infertility) OR (TI "reproductive sterility" OR AB "reproductive sterility") OR (TI subfertility OR AB subfertility)) OR ((MH "Cervix Neoplasms+") OR (TI "cervical neoplasm" OR AB "cervical neoplasm") OR (TI "cervical neoplasms" OR AB "cervical neoplasms") OR (TI "cervical cancer" OR AB "cervical cancer") OR (TI "cervix cancer" OR AB "cervix cancer")) OR ((MH "Human Papillomavirus Viruses+") OR (MH "Papillomavirus Infections+") OR (TI "Human Papilloma Virus" OR AB "Human Papilloma Virus") OR (TI "Human Papillomavirus" OR AB "Human Papillomavirus") OR (TI HPV OR AB HPV)) OR ((MH "Human Immunodeficiency Virus"+) OR (TI HIV OR AB HIV) OR (TI "Human Immunodeficiency Virus" OR AB "Human Immunodeficiency Virus") OR (TI AIDS OR AB AIDS) OR (TI "Acquired Immune Deficiency Syndrome Virus" OR AB "Acquired Immune Deficiency Syndrome Virus") OR (TI "Acquired Immunodeficiency Syndrome Virus" OR AB "Acquired Immunodeficiency Syndrome Virus")) OR ((MM "Sexually Transmitted Diseases+") OR (TI "sexually transmitted disease" OR AB "sexually transmitted disease") OR (TI "sexually transmitted diseases" OR AB "sexually transmitted diseases") OR (TI "sexually transmitted infection" OR AB "sexually transmitted infection") OR (TI "sexually transmitted infections" OR AB "sexually transmitted infections") OR (TI STD OR AB STD) OR (TI STI OR AB STI) OR (TI STIs OR AB STIs) OR (TI STDs OR AB STDs) OR (TI "venereal disease" OR AB "venereal disease") OR (TI "venereal diseases" OR AB "venereal diseases")) | 603,905 |
| #2 Artificial intelligence | ((MH "Artificial Intelligence+") OR (MH "Machine Learning+") OR (MH "Neural Networks (Computer)+") OR (TI "computer reasoning" OR AB "computer reasoning") OR (TI "machine learning" OR AB "machine learning") OR (TI "artificial intelligence" OR AB "artificial intelligence") OR (TI "computational intelligence" OR AB "computational intelligence") OR (TI "machine intelligence" OR AB "machine intelligence") OR (TI "computer learning" OR AB "computer learning") OR (TI "predictive analytics" OR AB "predictive analytics") OR (TI "gradient boosted decision" OR AB "gradient boosted decision") OR (TI "support vector machine" OR AB "support vector machine") OR (TI "random forest model" OR AB "random forest model") OR (TI "neural network" OR AB "neural network") OR (TI "neural networks" OR AB "neural networks") OR (TI "manifold learning" OR AB "manifold learning") OR (TI "deep learning" OR AB "deep learning") OR (TI "active learning" OR AB "active learning") OR (TI "learning algorithm" OR AB "learning algorithm") OR "learning algorithms" OR (TI "AI algorithms" OR AB "AI algorithms") OR (TI AI) OR (TI "natural language processing" OR AB "natural language processing") OR (TI "natural language understanding" OR AB "natural language understanding") OR (TI "software as a medical device" OR AB "software as a medical device") OR (TI chatbot OR AB chatbot) OR (TI "conversational agent" OR AB "conversational agent") OR (TI "large language model" OR AB "large language model") OR (TI "large language models" OR AB "large language models") OR (TI LLMs OR AB LLMs) OR (TI "sentiment analysis" OR AB "sentiment analysis") OR (TI "quantitative distribution" OR AB "quantitative distribution") OR (TI "frequency distribution" OR AB "frequency distribution")) | 54,521 |
| #3 Combined concepts | #1 AND #2 | 1,623 |
| #4 Combined with filters | #3 AND Filter: Source Types: Academic Journals | 1,507 |
